# Possible gift authorship and undeclared conflict of interest in: “Effect of self‐administration of medication programme on cardiovascular inpatients' medication adherence and nurses' satisfaction: A randomized clinical trial. *Nursing Open* 2021: 8(4):1947–1957.”

**DOI:** 10.1002/nop2.1209

**Published:** 2022-10-20

**Authors:** Richard Gray, David R. Thompson, Martin Jones, Dan Bressington

**Affiliations:** ^1^ 2080 School of Nursing and Midwifery La Trobe University Melbourne Vic. Australia; ^2^ 1067 Academic Department of Rural Health University of South Australia Adelaide SA Australia; ^3^ 10095 College of Nursing and Midwifery Charles Darwin University Darwin NT Australia; ^4^ School of Nursing and Midwifery Queens University Belfast UK

## CONFLICT OF INTEREST

The authors declare no conflicts of interest.

To the editor,

The effect on adherence of a self‐administration medication programme was tested in a randomized controlled trial involving 60 cardiac inpatients by Hajialibeigloo et al. (2021) and published in *Nursing Open*. In the trial treatment, adherence was determined using the Morisky Medication Adherence Scale (MMAS). According to the acknowledgement section of the paper, the trial was part of a master's degree thesis. The trial authors concluded that self‐administration was effective at increasing medication adherence. On reading the paper, one of the listed authors was Dr Morisky, who developed the MMAS, the stated primary outcome in this trial. It is unusual for the authors of a study measure to be included as an author, so our curiosity was aroused.

## 1 DR MORISKY AND THE MMAS

Dr Morisky has received attention over the last few years for asserting copyright over the MMAS. We counted at least six Retraction Watch posts related to Dr Morisky and his adherence scale (see, e.g. Marcus, 2021). There are also numerous case examples of researchers that have used the MMAS—without permission—receiving demands for money or immediate retraction of the paper: Hale et al. (2019) have reported a corrigendum and editorial warning about the MMAS. The authors and the *Journal of Medical Internet Research* editors were forced to issue the correction because of legal threats by Dr Morisky (Hale et al., 2019). The original paper reported the results of a trial of a remote medication monitoring system for heart failure patients, and the MMAS was one of the adherence measures used in the study (Hale et al., 2016). In the corrigendum, the MMAS was removed from the paper and an alternative adherence measure added. The editor warned researchers: “the developers of this scale are known to comb the literature and ask those who used the scale for research to pay for a retroactive license which may cost thousands or tens of thousands of dollars, and to add references to their work” (Hale et al., 2019).

## 2 WAS DR MORISKY GIFTED AUTHORSHIP?

Dr Morisky is listed as an author in the Hajialibeigloo et al. (2021) trial. Given his record as a researcher with an interest in adherence, it is plausible that Dr Morisky was a legitimate investigator on the study; however, this does not appear to be the case. According to the author contribution section of the paper, the study was designed by three researchers, and data collection and analysis were carried out by the first and second authors (Hajialibeigloo et al., 2021, page 1956). The authors make it clear that Dr Morisky was “not involved in any data analysis in this study” [sic] (Hajialibeigloo et al., 2021, page 1956). According to the author contribution statement, the only part of the research that Dr Morisky was involved in was manuscript preparation. This is potentially problematic as the ICMJE (International Committee of Medical Journal Editors) has a clear definition of what constitutes an author. According to the ICMJE to be considered an author a researcher must meet all of the following criteria: (1) Substantial contributions to the conception or design of the work; or the acquisition, analysis or interpretation of data for the work; AND (2) Drafting the work or revising it critically for important intellectual content; AND (3) Final approval of the version to be published; AND (4) Agreement to be accountable for all aspects of the work in ensuring that questions related to the accuracy or integrity of any part of the work are appropriately investigated and resolved (ICMJE, 2022). According to the ICMJE guidance, if all criteria are not met, their contribution should be noted in the acknowledgement section of the paper. In the (Hajialibeigloo et al., 2021) paper, the authors indicate that all authors “meet at least one” [sic] of the ICMJE criteria, which is seemingly a misinterpretation of the guidance. It appears Dr Morisky does not meet criteria for authorship. Further, evidence from a second paper seems to confirm that Dr Morisky was gifted authorship (Haji Ali Beigloo et al., 2019). In the second paper, Haji Ali Beigloo et al. (2019) seemingly report a separate “slice” of the trial (self‐efficacy, knowledge and satisfaction outcomes) but do not include Dr Morisky as an author. If Dr Morisky had contributed to the work in a manner that was consistent with ICMJE authorship guidelines for the first paper then he should have been included in the second.

## 3 CONFLICT OF INTEREST?

The authors of the Hajialibeigloo et al. (2021, page 1956) trial have declared “no conflict of interest” in the manuscript. As we have already noted, Dr Morisky developed the primary outcome used in this trial and has profited financially for its use in research. The ICMJE also has guidance on Disclosure of Financial and Non‐Financial Relationships and Activities, and Conflicts of Interest (ICMJE, 2022), stating that “when authors submit a manuscript of any type or format they are responsible for disclosing all relationships and activities that might bias or be seen to bias their work.” It seems to us that Dr Morisky has a conflict of interest that should have been declared.

## 4 WHY GIFTING AUTHORSHIP MATTERS

The gifting of authorship is common; authors of an observational study of three general medical journals found that up to 20% of papers reviewed had honorary authors (Bates et al., 2004). The Hajialibeigloo et al. (2021) paper is perhaps a rather unusual example of gift authorship because of Dr Morisky's involvement with the MMAS. Jones and McCullough (2015) have argued that unearned authorship should be considered a form of plagiarism because the author is passing off work that they have not done as their own. We see that the editors of *Nursing Open* also take authorship integrity seriously; it is why authors are asked to include an author contribution statement when submitting their manuscript.

It would be informative to understand from the trial authors why Dr Morisky was included as an author on their paper. If he made the required substantial contribution to the research, then the author contribution statement in the paper should be corrected, as should the conflict‐of‐interest section of the manuscript. Dr Morisky would then probably also then have a claim to authorship on the second paper (Haji Ali Beigloo et al., 2019). If Dr Morisky was gifted authorship, he should be removed as an author on this paper.

## 5 RECOMMENDATIONS

The Hajialibeigloo et al. (2021) trial may serve as a reminder that authorship should be earned and not gifted. Those in research leadership roles and journal editors have essential educational roles in ensuring the authors listed in papers have made a genuine and substantial contribution to the work. They should also have policies in place to ensure authors are aware of the ICJME requirements.

## Data Availability

Data sharing not applicable to this article as no data sets were generated or analysed during the current study.
